# Genetic Evolution of Avian Influenza A (H9N2) Viruses Isolated from Domestic Poultry in Uganda Reveals Evidence of Mammalian Host Adaptation, Increased Virulence and Reduced Sensitivity to Baloxavir

**DOI:** 10.3390/v14092074

**Published:** 2022-09-18

**Authors:** Gladys Atim, Titus Tugume, Qouilazoni A. Ukuli, Bernard Erima, Andrew Mubiru, Hannah Kibuuka, Edison Mworozi, Pamela McKenzie, Jasmine C. M. Turner, David Walker, Trushar Jeevan, Robert G. Webster, Jeremy Jones, Richard J. Webby, Mariette F. Ducatez, Fred Wabwire-Mangen, Denis K. Byarugaba

**Affiliations:** 1 Makerere University Walter Reed Project, Kampala P.O. Box 16524, Uganda; 2 St Jude Children’s Research Hospital, MS330, Memphis, TN 38105, USA; 3 Interactions Hôtes-Agents Pathogène, Ecole Nationale Vétérinaire de Toulouse, 31076 Toulouse, France; 4 School of Public Health, College of Health Sciences, Makerere University, Kampala P.O. Box 7062, Uganda; 5 College of Veterinary Medicine, Makerere University, Kampala P.O. Box 7062, Uganda

**Keywords:** influenza, phylogenetics, molecular markers, reassortment

## Abstract

A (H9N2) avian influenza A viruses were first detected in Uganda in 2017 and have since established themselves in live bird markets. The aim of this study was to establish the subsequent genetic evolution of H9N2 viruses in Uganda. Cloacal samples collected from live bird market stalls in Kampala from 2017 to 2019 were screened by RT-PCR for influenza A virus and H9N2 viruses were isolated in embryonated eggs. One hundred and fifty H9N2 isolates were subjected to whole genome sequencing on the Illumina MiSeq platform. The sequence data analysis and comparison with contemporary isolates revealed that the virus was first introduced into Uganda in 2014 from ancestors in the Middle East. There has since been an increase in nucleotide substitutions and reassortments among the viruses within and between live bird markets, leading to variations in phylogeny of the different segments, although overall diversity remained low. The isolates had several mutations such as HA-Q226L and NS-I106M that enable mammalian host adaptation, NP-M105V, PB1-D3V, and M1-T215A known for increased virulence/pathogenicity and replication, and PA-E199D, NS-P42S, and M2-S31N that promote drug resistance. The PA-E199D substitution in particular confers resistance to the endonuclease inhibitor Baloxavir acid, which is one of the new anti-influenza drugs. Higher EC50 was observed in isolates with a double F105L+E199D substitution that may suggest a possible synergistic effect. These H9N2 viruses have established an endemic situation in live bird markets in Uganda because of poor biosecurity practices and therefore pose a zoonotic threat. Regular surveillance is necessary to further generate the needed evidence for effective control strategies and to minimize the threats.

## 1. Introduction

Avian influenza viruses (AIVs) are negative-sense RNA viruses of the *Orthomyxoviridae* family whose natural hosts are birds [1]. They constantly undergo evolution mediated through mutation and reassortment resulting in viruses with varying pathogenicity, virulence, host specificity, antigenicity, and drug susceptibility [2]. The incorporation of mutations is driven by the influenza RNA polymerase which lacks proofreading functions [3]. Reassortment is a result of the segmented nature of the genome and mixing of genes following coinfections [3,4,5].

Of the diversity of viruses in avian hosts, A(H9N2) viruses are of particular interest. While being of low pathogenicity for birds, they have donated gene segments to novel influenza strains, such as H5N1, that appear to support infection of mammalian hosts including humans [6,7,8,9,10,11].

First isolated in the United States in 1966 [12], H9N2 viruses have been detected in many regions of the world and those belonging to the G1 lineage have been endemic in the Middle East (ME) since the late 1990s and in North Africa since the late 2000s. They were not, however, documented in sub-Saharan Africa until 2017, despite surveillance activities [13,14].

In Uganda, H9N2 viruses were first detected in poultry in 2017 and have since become established in live bird markets (LBMs) in part due to poor biosecurity [15]. A recent study found that H9N2 viruses from Uganda had reassorted more than those from West Africa [16], warranting further investigation.

Although they only cause mild to moderate illness in birds, H9N2 viruses impart a substantial economic burden by reducing productivity and performance of affected flocks. In addition, as of June 2022, there have been 102 laboratory-confirmed cases of human infection with H9N2 and 2 recorded deaths [17] in various countries including China [10,18], India [19,20], and Egypt [13], and the same in Senegal as of 2021; the human virus from Senegal was genetically similar to Ugandan H9N2 viruses [21]. In almost all reported human cases, previous contact with poultry was identified as a risk factor so H9N2 viruses remain a constant zoonotic threat [1,22]. In this study, we investigated H9N2 virus evolution in Uganda, identified further reassortment events and detected several mutations of interest.

## 2. Materials and Methods

### 2.1. Sample Collection

Cloacal–oropharyngeal samples were collected from birds in LBMs around Kampala city (Kalerwe, Kasubi, Nakasero and Nakawa markets which receive birds from all over the country) from 2017 to 2019. Samples were collected once a month on the same day, from up to 20 birds that were randomly selected from multiple stalls in each of the four markets. A total of 80 samples were collected each month. Samples were collected using Dacron swabs and stored in antibiotic enriched virus transport media.

RNA was extracted from swabs by using a QIAamp Viral RNA extraction kit (Qiagen, Germany) according to the manufacturer’s instructions, and they were tested for Influenza A by real time RT-PCR as previously described [16]. Positive samples were further tested for various subtypes including H9 subtype by RT-PCR.

Samples that were H9 positive were inoculated into 9-day old embryonated chicken eggs. These were incubated at 37 °C for at least 72 h. The eggs were then refrigerated for at least 4 h before allantoic fluid was harvested. 

### 2.2. Whole Genome Sequencing and Sequence Identification

Following a confirmatory PCR for H9, isolates with a CT value below 30 were selected for whole genome sequencing; in total, there were 150. RNA was extracted and all eight gene segments were amplified in an RT-PCR with SuperScript III One-Step RT-PCR System with Platinum^®^ Taq High Fidelity DNA Polymerase (Invitrogen, Waltham, MA, USA). Duplicate multiplexed paired-end sequencing libraries were generated using an Illumina Nextera XT library prep kit (FC-131-1096). The libraries were then fragmented and tagged with Illumina MiSeq-compatible barcode adapters using the NEBNext Ultra DNA Library Preparation kit (New England Biolabs, Ipswich, MA, USA) as previously described [16]. Paired-end sequencing was conducted on the Illumina MiSeq platform. Sequences were deposited into GenBank under accession numbers MW205009 to MW205198, MZ542846 to MZ543305, OP388450 to OP388679 and OP388789 to OP388827.

### 2.3. Sequence Analysis

Consensus sequences for each segment were generated. A BLAST search was conducted to find the most related sequences by E-score. In order to determine whether the isolates carried any specific Sequence Features (SF) of importance, SF variant type analysis was performed in the Influenza Research Database (IRD) [23].

Groups of sequences were aligned using the MUSCLE alignment tool offered in the MEGA11 software along with calculations of maximum composite likelihood, pairwise and overall mean distances [24]. Phylogenetic analysis was performed using BEAST v1.10.4. This was carried out with a relaxed (uncorrelated lognormal) molecular clock for a constant population size coalescent tree prior. Other pre-set conditions were a Hasegawa–Kishino–Yano (HYK) + Gamma (4 categories) nucleotide substitution model, a consideration of 3 codon positions and an unlinked base frequency across all codon positions. The length of Markov chain Monte Carlo (MCMC) was set at 100 million states.

The output was assessed in Tracer v1.7.1 to determine various evolution parameters such as the annual mean rate of nucleotide substitution per site in the segments, their time to most common recent ancestor (tMRCA) as well as the number of lineages through time. Maximum Clade Credibility (MCC) trees with a 10% burn-in were generated using the TreeAnnotator v1.10.4 and visualized using FigTree v1.4.4 [25].

### 2.4. Assessment of H9N2 Susceptibility to Baloxivir (BXA)

Full plaque reduction EC50 analysis was performed on randomly selected PA-E199D H9N2 viruses to determine susceptibility to BXA. MDCK cells (1 × 10^6^) were plated in six-well plates, inoculated with influenza viruses (multiplicity of infection [MOI] of 0.001), cultured in MEM containing 1% bovine serum albumin (BSA), 1 μg/mL TPCK trypsin, and BXA (0, 1, 10, or 100 nM). At 1 h post-virus inoculation, cells were washed, overlaid with MEM containing 0.45% immunodiffusion-grade agarose (MP Biomedicals, Santa Ana, SC, USA), 1% BSA, 1.8 μg/mL TPCK trypsin, and BXA (0.001 nM to 500 nM). At 72 hpi to 120 hpi, the overlays were removed, and the cell monolayers were stained with 1% crystal violet–10% formaldehyde. The number of PFUs per well was calculated, and EC_50_s were determined by using the log (inhibitor) versus response logistic nonlinear regression equation in GraphPad Prism 8.0 software.

### 2.5. Hemagglutination Inhibition (HI) Assay

Antigenic analysis was also carried out on the selected viruses with additional antigens and serum samples that were provided by members of the World Health Organization Global Influenza Surveillance and Response System. A total of 25 µL of PBS was dispensed into each well of a 96-well microtiter plate. A total of 25 µL of reference anti-serum was added to the first well the plate and two-fold dilutions made. Four hemagglutinating units of virus was added to each well and left for a minimum of 30 min at room temperature, then 0.5% (*v*/*v*) turkey RBCs were added to each well and allowed to settle for 40 min at room temperature. The HI titer recorded was the highest dilution of serum causing complete inhibition of hemagglutination [26].

## 3. Results

### 3.1. H9N2 Detection

A total of 2670 samples were collected between 2017 and 2019. The overall prevalence of H9N2 virus was 49%, with a steady increase from 47% in 2017 to 61% in 2019. Positivity rates in individual markets ranged from 33% to 68% [16].

### 3.2. Phylogenetic Analyses

**Figure 1:**
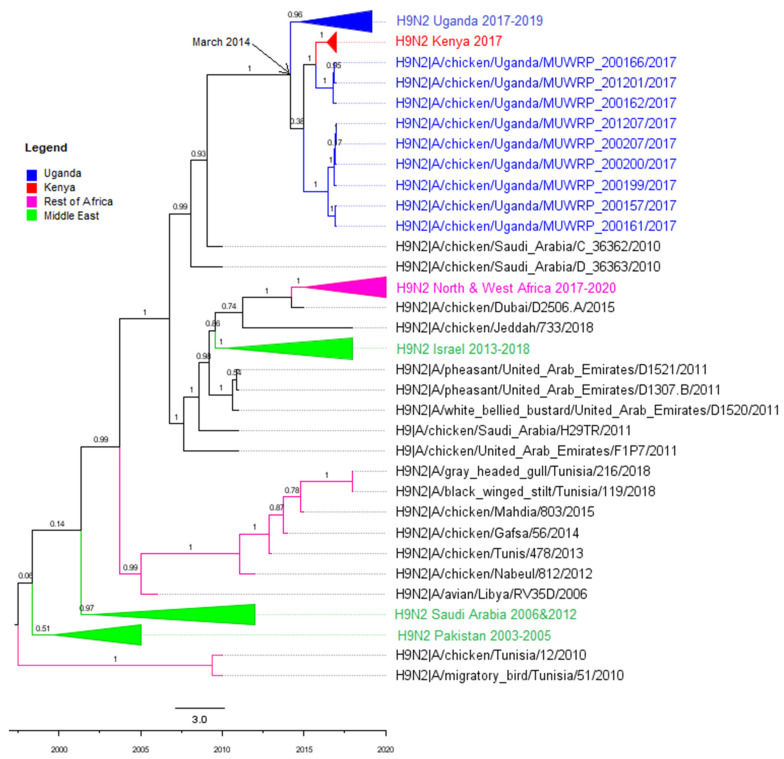
Phylogenetic tree for HA. Ugandan viruses are highlighted in blue, Kenyan in red, North and West African countries in pink, and the Middle East in the green clade. Posterior probabilities are shown on the branches. The top scale represents the estimated number of substitutions and the bottom scale shows time of divergence. HKY + G (4) nucleotide substitution model and an MCMC of 100 million states was applied in BEAST to generate this tree.

In total, 150 isolates were selected for whole genome sequencing. Of these, 48 were from Nakawa market, 42 from Nakasero market, 25 from Kalerwe market, and 35 from Kasubi market. All were from chicken and belonged to the G1 lineage. Phylogenetic analysis of the HA segment showed that the Uganda H9N2 viruses clustered in the same genetic clade as viruses from Kenya, (Figure 1) and were similarly related to viruses from Saudi Arabia, the United Arab Emirates and those from Western and Northern Africa: Algeria, Benin, Burkina Faso, Ghana, Libya, Morocco, Senegal, Togo and Tunisia.

**Figure A1:**
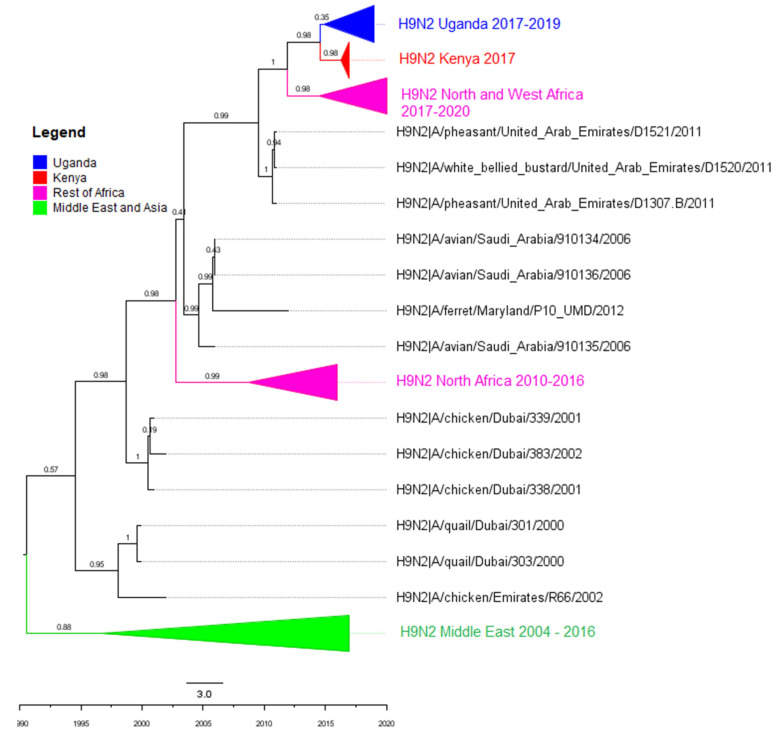
Phylogenetic tree for PB2. Ugandan viruses are highlighted in blue, Kenyan in red, North and West African countries in pink, and the Middle East in the green clade. Posterior probabilities are shown on the branches. tMRCA for Uganda is November 2014. Scales represent substitutions and time.

**Figure A2:**
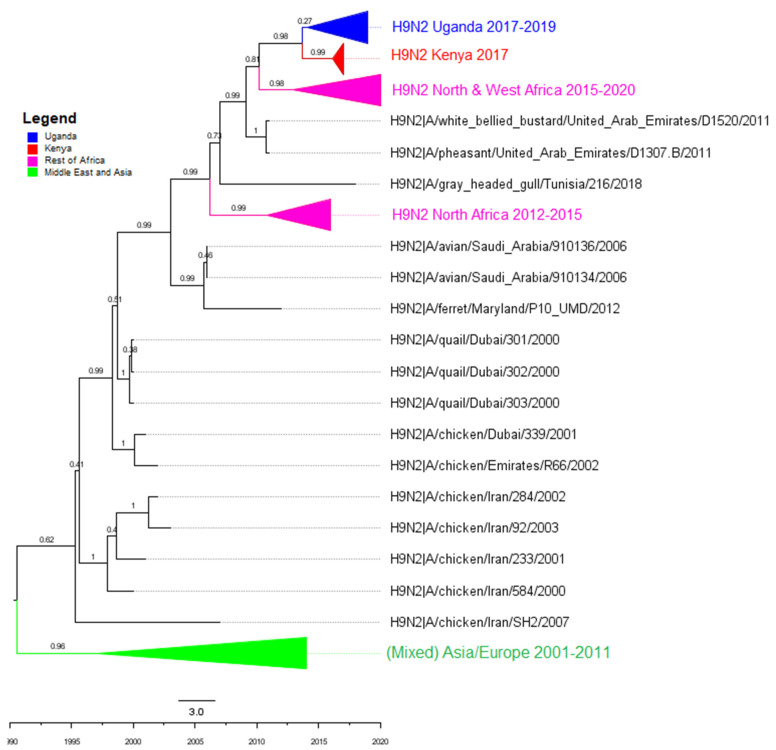
Phylogenetic tree for PB1. Ugandan viruses are highlighted in blue. Kenyan in red, North and West African countries in pink, and the Middle East, Asia and a few European viruses in the green clade. tMRCA for Uganda is August 2013. Posterior probabilities are shown on the branches. Scales represent substitutions and time.

**Figure A3:**
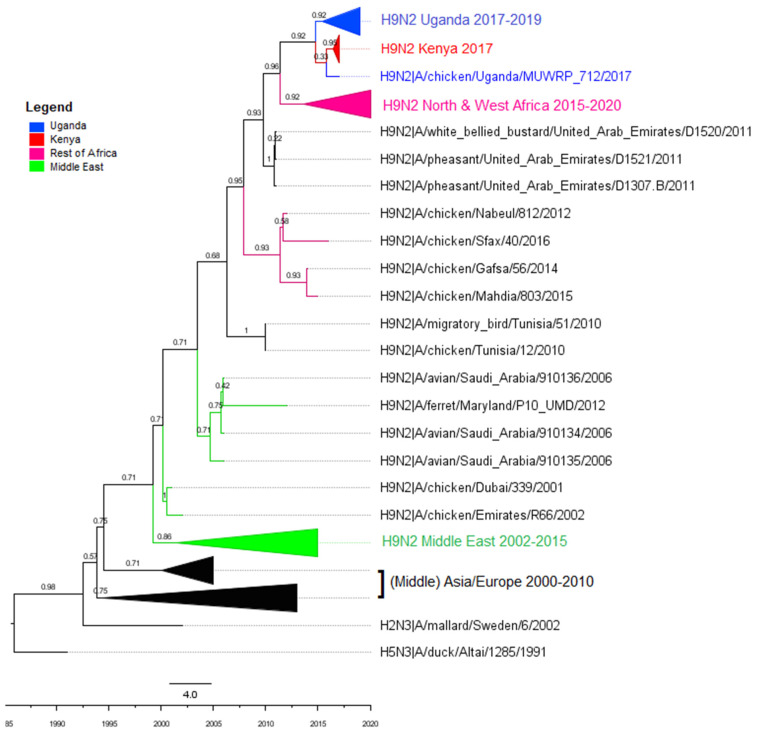
Phylogenetic tree for PA. Ugandan viruses are highlighted in blue, Kenyan in red, North and West African countries in pink, and the Middle East in the green clade Including various non H9N2 viruses from Asia and Europe tMRCA for Uganda is September 2014. Posterior probabilities are shown on the branches. Scales represent substitutions and time.

**Figure A4:**
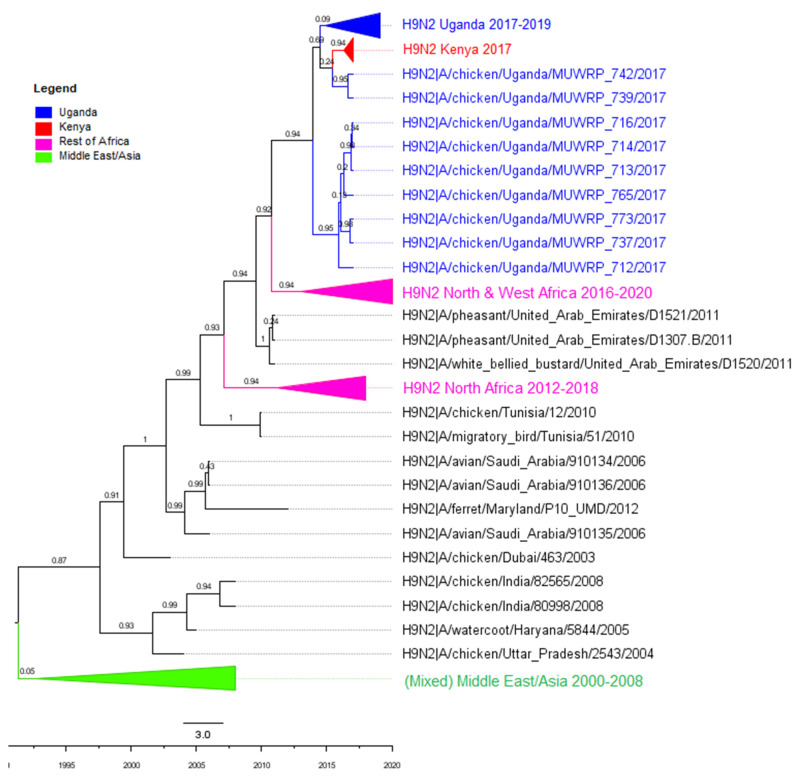
Phylogenetic tree for NP. Ugandan viruses are highlighted in blue, Kenyan in red, North and West African countries in pink, and the Middle East and Asia in the green clade. tMRCA for Uganda is December 2013. Posterior probabilities are shown on the branches. Scales represent substitutions and time.

**Figure A5:**
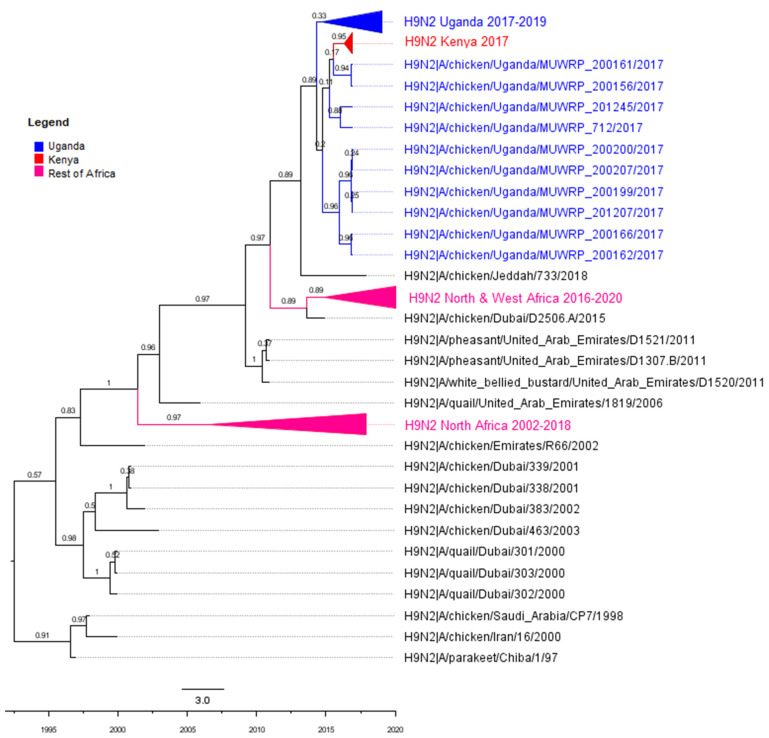
Phylogenetic tree for NA. Ugandan viruses are highlighted in blue, Kenyan in red, North and West African countries in pink. tMRCA for Uganda is May 2014. Posterior probabilities are shown on the branches. Scales represent substitutions and time.

**Figure A6:**
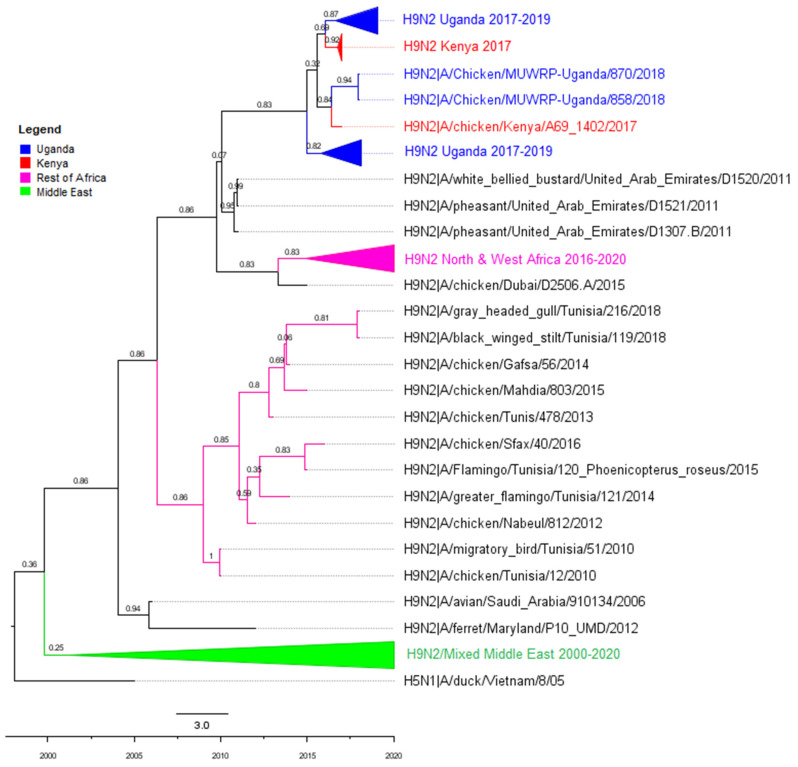
Phylogenetic tree for MP. Ugandan viruses are highlighted in blue, Kenyan in red, North and West African countries in pink, and the Middle East in the green clade. tMRCA for Uganda is December 2014. Posterior probabilities are shown on the branches. Scales represent substitutions and time.

**Figure A7:**
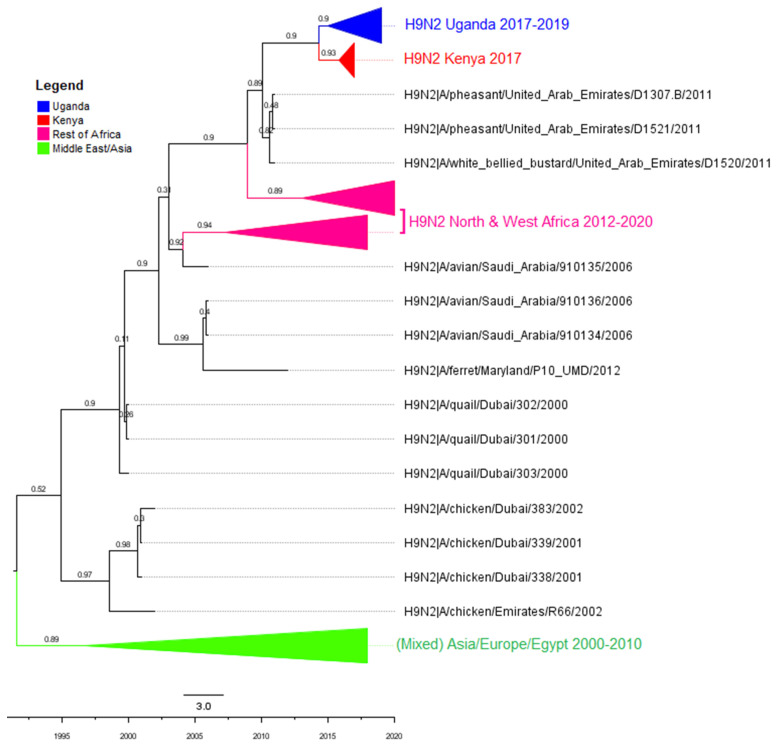
Phylogenetic tree for NS. Ugandan viruses are highlighted in blue, Kenyan in red, North and West African countries in pink, and the Middle East and Asia in the green clade. tMRCA for Uganda is May 2014.Posterior probabilities are shown on the branches. Scales represent substitutions and time.

The other gene segments had similar patterns of relatedness as the HA as shown in Figure A1, Figure A2, Figure A3, Figure A4, Figure A5, Figure A6 and Figure A7 (Appendix A) of other segments, respectively. The segments had very similar trees with no major differences in the sequences they were most closely related to. There were, however, minor differences in clade arrangement and general tree topology.

### 3.3. Molecular Clock Analysis

The tMRCA of the Uganda H9N2 HA gene as calculated by BEAST was March 2014 (interval from 2013 to 2015 for 95% of the samples). The tMRCA range for all the segments also fell within the same time range of 2013 to 2014. It was, however, difficult to pinpoint a true ancestor as the most closely related sequences (from A/Pheasant/United_Arab_Emirates/D1307/2011) were quite distant from the Uganda clade in terms of branch length and isolation date. This suggests a gap in surveillance over this time period.

**Figure 2:**
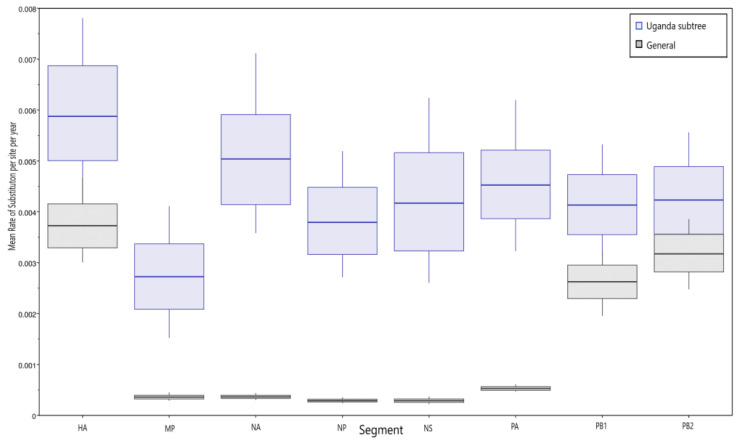
Comparing the mean rate of substitutions of the segments in Ugandan viruses and the rest. Ugandan viruses had a higher-than-average substitution rate.

**Figure 3:**
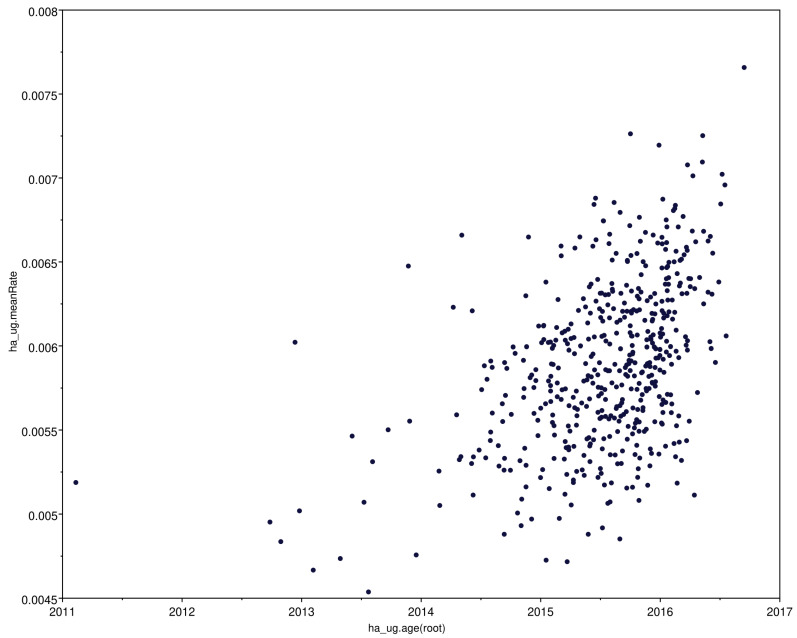
Mean rate of nucleotide substitution per site in the HA segment of Uganda H9N2 viruses over time. Each dot represents a branch in the tree and the age(root) corresponds to time of divergence.

The genetic diversity of the Ugandan viruses calculated by maximum composite likelihood ranged from 0.02% to 0.1%. The annual mean rate of substitutions per site was higher overall in the Ugandan virus subtree than in the rest of the H9N2 viruses with the HA segment having the most substitutions, while the MP had the lowest under the uncorrelated lognormal clock (Figure 2). When plotted against time, there was evidence of an increasing substitution rate as shown for the HA segment (Figure 3).

### 3.4. Reassortment

**Figure 4:**
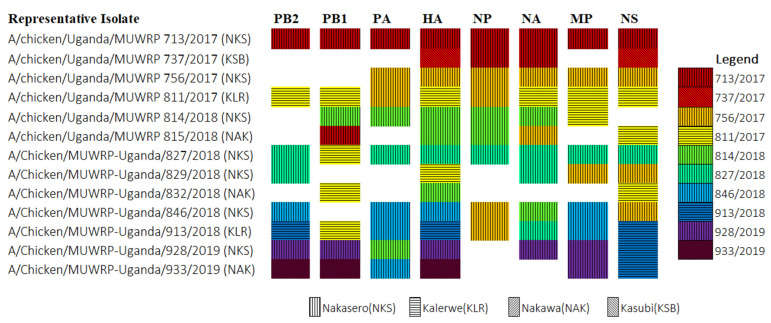
Reassortment within Ugandan H9N2 viruses. Clades were created in each segment by collapsing all branches with length < 1. From these, the oldest virus isolate was selected and those from the HA segment were given a color code. Representative isolates were cross-referenced to see if they fell within the same clades across all segments. Symbols for markets of origin were also assigned and overlaid.

Pairwise distances between the Ugandan H9N2 viruses gave rise to multiple clusters in different gene segments; however, the specific clustering differed from one segment to another and did not segregate by LBM of origin. This was also evident in the tree topology with multiple sub-clades within the Ugandan clusters that differed from segment to segment. Together, these findings are consistent with reassortment between H9N2 viruses within Uganda. To investigate this possibility, all clades containing branches of lengths < 1 were collapsed, and a representative taxon (the oldest) was picked from each clade. The clades in HA were used as points of reference and color-coded in creating Figure 4, to visualize reassortment events. All twelve representative isolates had unique constellations (different patterns). Only two isolates, A/Chicken/Uganda/MUWRP/713/2017 and A/Chicken/Uganda/MUWRP/756/2017, showed no reassortment at all. Besides HA (the reference segment), the PB2 segment had the most reassortment, with the greatest number of unique clades, while the NP segment had the least reassortment, with only four clades, all from the same market. This complexity is consistent with introduction of genetic diversity from different markets. These findings suggest that there is a significant level of reassortment within and between the different LBMs in our study.

### 3.5. Molecular Marker Analysis

A detailed examination of sequence features within the Ugandan H9N2 virus sequences revealed multiple substitutions in the H9N2 viruses that are associated with increased virulence in both mammals and avian species, increased virus replication, and reduced sensitivity to antiviral drugs. Details of the relevant substitutions in the different segments found in each segment are presented in Table 1.

Among the sequence feature variants identified were those that have been associated with infection in mammals such as Q226L in HA, which increases binding to sialic acid receptors preferred by mammalian-adapted viruses [27]. Others that increase virulence in mammals were N30D and T215A in M1 [28] and NS-P42S [29].

Multiple mutations that are known to increase replication via enhanced polymerase activity were also found and included NP-E210D, S37A and N383D in PA, D3V and D622G in PB1, D9N and K226R in PB2 [30,31,32,33,34,35].

**Figure 5:**
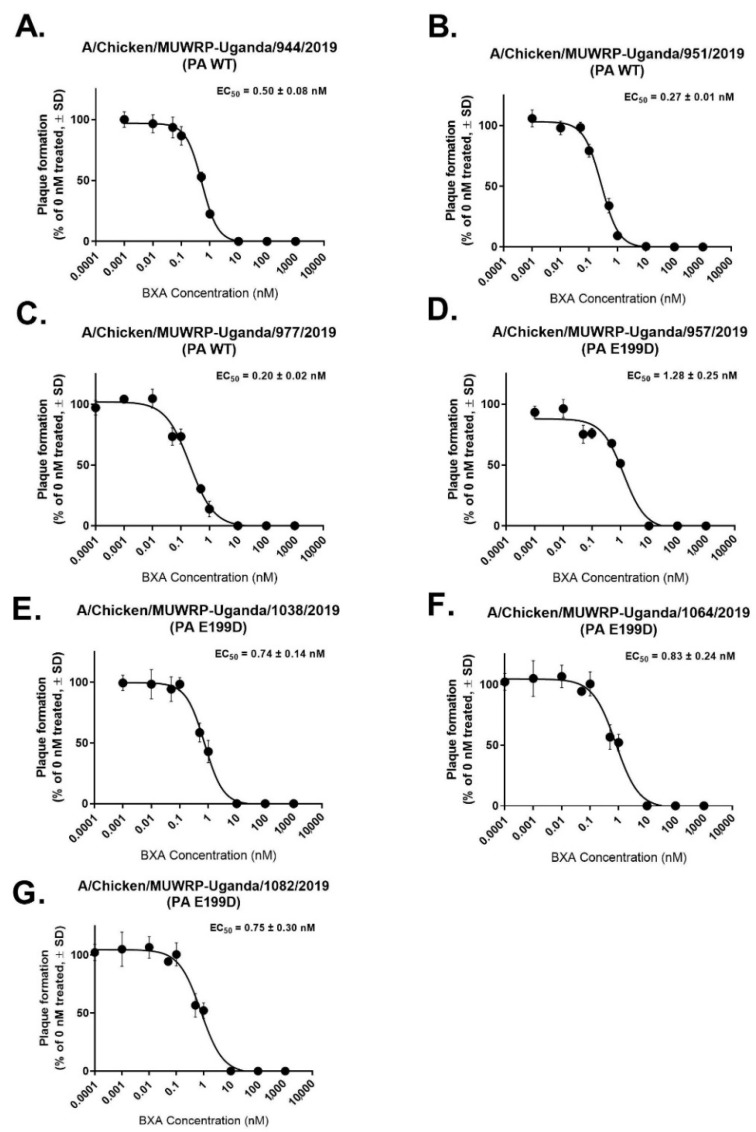
BXA Susceptibility of H9N2 viruses from Uganda containing the PA E199D substitution. The wild type (WT) viruses in (**A**–**C**) have EC50s ranging from 0.2–0.5 nM while those with the ED substitution (**E**–**G**) have increased EC50s of 0.74–0.83 nM. (**D**) shows a virus which had both PA F105L+E199D had the highest EC50 of 1.28nM, which suggests a synergistic effect. Data is representative of an average of three independent dose-response curves.

Sequence markers associated with increased resistance to amantadine were also present in M2 (S31N) [36]. Eight H9N2 viruses were found to have a PA-E199D mutation, which is a potential marker associated with reduced sensitivity to Baloxivir (BXA) [37]. Wild-type (WT) E199 and E199D viruses were tested for their susceptibility to BXA. An almost three-fold reduction in the median EC50 value was observed for H9N2 viruses with the E199D mutation (0.7–1.2 nM for E119D compared to 0.1–0.5 nM for WT) (Figure 5).

### 3.6. Antigenic Analysis

Selected viruses were evaluated for their antigenic properties using a panel of reference antigens and antisera. The selected viruses were antigenically similar to each other and reacted within four-fold to post-infection antiserum raised against A/Oman/2747/2019 IDCDC-RG66A, a human H9N2 virus (Table 2).

## 4. Discussion

Non-human Influenza A viruses were first reported in Uganda during a country-wide survey carried out in 2014 [41] with a prevalence of 1.1% in livestock and poultry. Regular surveillance did not reveal any remarkable findings until 2017 when H9N2 viruses were first documented in LBMs. As a consequence of this finding, we conducted monthly surveillance in four LBMs from 2017 to 2019 as previously reported [16]. It is possible that infections are amplified in LBMs due to poor biosecurity and management practices that foster virus maintenance [15]. Ugandan LBMs receive birds from different sources, have insufficient cleaning practices, and have no all-in-all-out policy with new birds introduced into cages where unsold birds are kept. These practices create an environment suitable for AIV maintenance, amplification, and evolution that consequently becomes a source of potential zoonotic viruses [42,43].

Genetic analysis of the Uganda H9N2 viruses isolated during our studies showed that they belong to the G1 lineage, more related to Kenyan viruses than to viruses from Northern and Western Africa. The Ugandan viruses also clustered with viruses from the Middle East and Asia (Saudi Arabia, Pakistan, Iran and United Arab Emirates). Unfortunately, few countries in Eastern, Central and Southern Africa conduct regular surveillance for AIVs, making it difficult to accurately determine the spread of viruses across the continent and identify ancestral viruses. We can, however, infer from the available data that H9N2 viruses in Kenya and Uganda most likely originated from the Middle East via poultry movement or human activities. Viruses were introduced into Uganda around 2014 and became firmly established in commercial markets by 2017.

The overall genetic diversity in each segment of the Ugandan H9N2 viruses was less than 0.5%. This is likely influenced by the limited geographic distribution of the markets we sampled, where spread might be localized and not indicative of overall diversity across the country. Nevertheless, the nucleotide substitution rate within the H9N2 viruses has been steadily increasing, and pairwise distance analysis revealed clusters within the different gene segments that showed a random pattern of distribution by market. The topologies of the Ugandan H9N2 sequences within the phylogenetic trees differed by segment as did the rate of substitution. This demonstrates a differing pattern of evolution in the gene segments in addition to segment-specific differences in rates of nucleotide substitution.

A major driver for evolution and risk of influenza viruses lies in reassortment. Over the years, multiple novel influenza A reassortants have been discovered that are more virulent and can infect multiple species [44,45,46]. In order for reassortment to happen, there must be co-infection within the host cells [4]. This can be between viruses of different strains or subtypes, or viruses from different locations or host species [46]. In this case, we investigated reassortment within the isolates and reassortment by market, and found significant levels in both instances. Cocirculation of highly pathogenic AIV in the same population has been demonstrated to provide an opportunity for these viruses to reassort as has been observed with H5N1 in Cambodia [47] and in Egypt [44,45,48]. With H6N2 viruses having recently been discovered in Uganda [49], monitoring for reassortment must be intensified. Consistent with the limited genetic diversity in HA, selected H9N2 viruses were antigenically similar to each other. In addition, they were antigenically similar to H9N2|A/Oman/2747/2019, a virus responsible for a recent human infection [19].

Molecular markers that have been reported to increase mammalian host susceptibility were also found in the Ugandan H9N2 viruses. These included the HA Q226L (H3 numbering) substitution which changes the flexibility of the 220-loop allowing it to interact more favorably with receptors on mammalian cells [50,51] and also promoting immune escape [52,53]. Also present in the HA were T197/G520 (H9 numbering), which have been associated with increased transmission of H9N2 viruses via respiratory droplets [54,55]. The canonical PB2 E627V/K mutations, responsible for increased virulence and adaptation in mice [56,57] were absent; however, this is compensated for by the presence of VDKGVV substitutions at positions 89, 309, 339, 477, 495 and 676 respectively [58,59].

Mutations that increase drug resistance against antivirals such as Amantadine and Baloxavir, which are the drugs of choice for treating severe Influenza infections, were also found in the MP and PA segments. This was in addition to mutations that increased pathogenicity in avian and mammalian cell lines, and in experimental animals such as ferrets and mice.

Our H9N2 viruses with PA-E199D demonstrated marginally lower susceptibility to the endonuclease inhibitor Baloxavir acid as has been reported for other viruses [37]. The virus with the combined F105L+E199D substitution had the highest EC50 which suggests a possible synergistic effect warranting further investigation. Many of the markers identified in the Ugandan viruses seem to be widespread in Africa, having been found in viruses from Kenya, Tunisia, and Algeria [60,61,62].

## 5. Conclusions

Our findings indicate that G1-lineage H9N2 viruses were introduced into Uganda from the Middle East around 2014. The viruses subsequently became established, quickly reaching a prevalence of 49%. Through comparative whole genome analysis, the viruses were found to have evolved extensively through reassortment events within and between the LBMs in segment-specific manners. There were also multiple molecular markers identified that have been associated with cross species infection; these include HA Q226L and those that enhance pathogenicity and reduce antiviral drug sensitivity. The documented zoonotic capacity of G1-lineage H9N2 viruses highlights the need for regular and exhaustive surveillance of all influenza viruses in circulation within the country and subsequent risk-assessment to provide a complete picture of prevalence, distribution, and risk.

## Figures and Tables

**Table 1:** Sequence Feature Variants in H9N2 virus proteins and their phenotypic function.

Protein	Sequence Features	Phenotype	Frequency
HA	T197, G520	Acquisition of respiratory droplet transmission and human-like clinical symptoms	All
	Q226L	Increased virus binding to α2–6, enhanced replication in mammalian cells and ferrets, enhanced contact transmission in ferrets	
M1 M2	N30D, I43M, T215A	Increased virulence in mice	S31N absent in 1
	S31N	Increased Amantadine resistance	
NP	M105V, A184K	Increased virulence in chicken [38,39]	All
	E210D	Increased polymerase activity in mammalian cell line	
NS	P42S	Increased virulence and decreased antiviral response in mice	All
	I106M	Increased viral replication in mammalian cells and virulence in mice [40]	
PA	S37A, N383D	Increased polymerase activity in mammalian and avian cell line	All E199D in 8
	E199D	Decreased sensitivity to Baloxivir	
PB1	D3V	Increased polymerase activity and replication in mammalian and avian cell lines	All
	D622G	Increased polymerase activity in mammalian cell lines	
PB2	L89V, G309D, T339K, R477G, I495V, A676V	Increased polymerase activity in mammalian cell line and increased virulence in mice	All
	D9N	Increased virulence in mice	
	K256R	Increased polymerase activity in mammalian cell lines	

**Table 2:** Hemagglutination inhibition assay of recent H9N2 viruses from Uganda (0.5% turkey RBC).

		Clade	Oman/2747	Bd/0994	HK/1073	HK/33982	qa/Bd/19462	ck/Benin	ck/Bd/46240	ck/BD/46129	Collection Date	Passage History
**Reference antigen**												
1	A/Oman/2747/2019 IDCDC-RG66A	G1	2560	320	<	40	640	320	640	640		V1E2/E1
2	A/Bangladesh/0994/2011-IDCDC-RG31	G1	1280	1280	<	80	640	640	1280	2560		V1E2/E1
3	A/Hong Kong/1073/99	G1	<	<	160	640	<	<	<	<		C4
4	A/Hong Kong/33982/2009-PR8-IDCDC-RG-26	G1	<	<	160	1280	<	<	<	<		V1E3/E2/E1
5	A/quail/Bangladesh/19462/2013	G1	320	40	10	80	320	80	160	160		E2
6	A/chicken/Benin/19-A-01-145-E/2019	G1	1280	320	<	80	640	640	640	1280		E1/E2
7	A/chicken/Bangladesh/46240/2020	G1	80	80	<	40	80	160	640	1280		E1
8	A/chicken/Bangladesh/46129/2020	G1	640	640	<	80	640	320	1280	2560		E1
**Test antigens**												
9	A/chicken/MUWRP-UGANDA/944/2019	G1	1280	2560	<	20	640	320	1280	2560	03/13/19	X/E1
10	A/chicken/MUWRP-UGANDA/951/2019	G1	2560	2560	<	40	1280	640	1280	2560	03/13/19	X/E1
11	A/chicken/MUWRP-UGANDA/957/2019	G1	1280	1280	<	20	320	320	1280	2560	04/24/19	X/E1
12	A/chicken/MUWRP-UGANDA/977/2019	G1	1280	1280	<	20	640	320	1280	2560	06/19/19	X/E1
13	A/chicken/MUWRP-UGANDA/1038/2019	G1	640	1280	<	20	320	320	640	1280	09/25/19	X/E1
14	A/chicken/MUWRP-UGANDA/1064/2019	G1	1280	1280	10	20	640	320	1280	2560	11/20/19	X/E1
15	A/chicken/MUWRP-UGANDA/1082/2019	G1	1280	1280	10	10	640	320	2560	2560	12/19/19	X/E1
Serum production: P = prime			CDC	P	P	P	P	P	P	P		

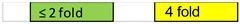
.

## Data Availability

Sequences analyzed in this work can be found in the GeneBank under accession numbers MW205009 to MW205198, MZ542846 to MZ543305, OP388450 to OP388679 and OP388789 to OP388827.
